# Proteomic Analysis of Plasma-Purified VLDL, LDL, and HDL Fractions from Atherosclerotic Patients Undergoing Carotid Endarterectomy: Identification of Serum Amyloid A as a Potential Marker

**DOI:** 10.1155/2013/385214

**Published:** 2013-12-24

**Authors:** Antonio J. Lepedda, Gabriele Nieddu, Elisabetta Zinellu, Pierina De Muro, Franco Piredda, Anna Guarino, Rita Spirito, Franco Carta, Francesco Turrini, Marilena Formato

**Affiliations:** ^1^Dipartimento di Scienze Biomediche, University of Sassari, Via Muroni 25, 07100 Sassari, Italy; ^2^Servizio di Chirurgia Vascolare, Clinica Chirurgica Generale, University of Sassari, Viale S. Pietro 43/B, 07100 Sassari, Italy; ^3^Centro Cardiologico “F. Monzino,” IRCCS, University of Milan, Via Carlo Parea 4, 20138 Milan, Italy; ^4^Nurex S.r.l., Via Predda Niedda Strada 3, 07100 Sassari, Italy; ^5^Dipartimento di Genetica, Biologia, Biochimica, University of Turin, Via Santena 19, 10126 Turin, Italy

## Abstract

Apolipoproteins are very heterogeneous protein family, implicated in plasma lipoprotein structural stabilization, lipid metabolism, inflammation, or immunity. Obtaining detailed information on apolipoprotein composition and structure may contribute to elucidating lipoprotein roles in atherogenesis and to developing new therapeutic strategies for the treatment of lipoprotein-associated disorders. This study aimed at developing a comprehensive method for characterizing the apolipoprotein component of plasma VLDL, LDL, and HDL fractions from patients undergoing carotid endarterectomy, by means of two-dimensional electrophoresis (2-DE) coupled with Mass Spectrometry analysis, useful for identifying potential markers of plaque presence and vulnerability. The adopted method allowed obtaining reproducible 2-DE maps of exchangeable apolipoproteins from VLDL, LDL, and HDL. Twenty-three protein isoforms were identified by peptide mass fingerprinting analysis. Differential proteomic analysis allowed for identifying increased levels of acute-phase serum amyloid A protein (AP SAA) in all lipoprotein fractions, especially in LDL from atherosclerotic patients. Results have been confirmed by western blotting analysis on each lipoprotein fraction using apo AI levels for data normalization. The higher levels of AP SAA found in patients suggest a role of LDL as AP SAA carrier into the subendothelial space of artery wall, where AP SAA accumulates and may exert noxious effects.

## 1. Introduction

Cardiovascular diseases are the leading cause of death and illness in developed countries, with atherosclerosis being the most important contributor. Atherosclerosis is a chronic inflammatory condition that could turn into an acute clinical event due to plaque rupture and thrombosis [1]. Indeed, vascular inflammation not only plays a major role in the development of atherosclerosis but also contributes to the acute onset of thrombotic complications [2]. The selective retention of circulating apolipoprotein B100 containing lipoproteins in the subendothelial space, by means of specific interactions with artery wall proteoglycans, is currently thought to be the leading event in atherogenesis [3, 4].

Lipoproteins are supramolecular complexes that deliver insoluble lipids from the tissues where they are synthesized to those that metabolize or store them. They consist of hydrophobic molecules (core), particularly triacylglycerol and cholesteryl esters, stabilized by a coat of amphipathic compounds, namely, phospholipids, unesterified cholesterol, and proteins, with the latter referred to as apolipoproteins (apo) [5]. Apolipoproteins are a very heterogeneous protein family implicated in lipoprotein stabilization, lipid metabolism, inflammation, or immunity [6]. Except for apo B100, the main structural apolipoprotein of VLDL and LDL, they may be exchanged among circulating lipoprotein classes during their physiological metabolism or in pathological conditions. For a long time now, lipoproteins have attracted a great deal of interest because of their implication in atherogenesis. Although it is well known that high LDL-cholesterol and low HDL-cholesterol levels are associated with increased risk for the development of cardiovascular disease, clinical studies suggest that levels of apo B100 and apo AI may be better predictors [7]. Since the protein component of these particles is largely responsible for carrying out their various functions, detailed information about the apolipoprotein composition and structure may contribute to revealing their role in atherogenesis and to developing new therapeutic strategies for the treatment of lipoprotein-associated disorders. Applying proteomics to the study of lipoproteins, including gel-based or gel-free technologies, may significantly contribute to the achievement of this goal. Indeed, recent proteomic studies have revealed that lipoproteins carry an array of proteins previously unexpected [8].

As far as we know, about thirty proteomic studies on VLDL, LDL, and HDL have been published up to now, while no proteomic studies on chylomicrons are reported in the literature [8]. Recently, the proteome of lipoprotein (a) has been investigated too [9]. The majority of these studies focused on characterizing the apolipoprotein cargo of the different lipoprotein fractions in healthy subjects. Conversely, only few studies dealt with differential apolipoprotein expression in relation to cardiovascular diseases. In this respect, HDL fraction has been the most studied, in association with coronary artery disease (CAD) [10–13], high cardiovascular risk and acute myocardial infarction [14], hyperlipidemia [15], or low levels of HDL cholesterol [16, 17]. One study on LDL fraction from atherosclerotic patients with the metabolic syndrome and diabetes [18] and one on VLDL fraction from hyperlipidemic subjects [19] were conducted. Several apolipoprotein isoforms involved in lipid metabolism, inflammation or immunity were detected as differentially expressed. Regrettably, these studies merely focused on one lipoprotein class at a time and were based on a low sample size.

Recently, by applying proteomics to the study of carotid plaque vulnerability, we identified a panel of proteins differentially expressed/oxidized in stable and unstable lesions [20, 21]. In the present study we set up a method for characterizing the exchangeable apolipoprotein component of plasma VLDL, LDL, and HDL fractions from patients undergoing carotid endarterectomy, useful for identifying differentially expressed proteins with respect to healthy normolipidemic subjects. By means of two-dimensional electrophoresis (2-DE) and MALDI-TOF MS analysis we identified acute-phase serum amyloid A protein (AP SAA) as overexpressed in the three lipoprotein classes from patients. Results validation was performed by western blotting analysis. In our knowledge, this is the first study providing information on exchangeable apolipoprotein profiles in VLDL, LDL, and HDL fractions from atherosclerotic patients undergoing carotid endarterectomy.

## 2. Materials and Methods

### 2.1. Sample Collection

Analyses were performed on 4 pooled plasma samples from 57 healthy normolipidemic volunteers and 4 pools obtained from 79 patients undergoing carotid endarterectomy. Carotid atherosclerosis was assessed by ultrasonography using a Mylab 70 X vision echocolor Doppler equipped with an LA332 AppleProbe 11–3 MHz (Esaote). All patients selected for surgery had either a high-grade stenosis (>70%) or an ulcerated lesion of a medium grade based on echo-Doppler analysis. All patients with hypertension, dyslipidemia, and/or diabetes were under pharmacological treatment. The main clinical parameters of patients and controls are summarized in Table 1. Informed consent was obtained before enrolment. Institutional Review Board approval was obtained. The study was conducted in accordance with the ethical principles of the current Declaration of Helsinki.

Fasting blood samples were collected into Vacutainer tubes containing EDTA and immediately centrifuged at 2000 ×g for 10 minutes at 4°C. Plasma was added with 100 *μ*M APMSF (4-amidinophenylmethane-sulfonylfluoride), 2 *μ*g/mL Kallikrein Inactivator (cyclohexylacetyl-Phe-Arg-Ser-Val-Gln amide), and 50 *μ*M leupeptin (Acetyl-Leu-Leu-Arg-Hydrochloride) and stored at −80°C until analysis. Before lipoprotein purification, equal amounts of plasma samples were randomly combined to yield 4 homogeneous pooled VLDL, LDL, and HDL fractions from both patients and controls.

### 2.2. Lipoproteins Purification

Lipoproteins were isolated by isopycnic ultracentrifugation according to the methods described by Himber et al. [22] and McDowell et al. [23] with slight modifications. Briefly, 3.9 mL of pooled plasma samples was added with NaBr to *d* = 1.3 g/mL (472.2 mg NaBr/mL plasma) in centrifuge tubes (Beckman Coulter, Thin Wall Ultra-Clear, 14 mL, 14 × 95 mm), overlaid with 8.1 mL of a 0.6% NaCl solution (*d* = 1.006 g/mL), and centrifuged at 285,000 ×g for 48 h at 4°C in an Optima L90 series ultracentrifuge equipped with a SW40 Ti rotor (Beckman Coulter). Afterwards, VLDL, LDL, and HDL fractions were collected and further purified by a second centrifugation step, performed at 285,000 ×g for 24 h in saline solutions at density 1.006, 1.063, and 1.21 g/mL, respectively. Lipoprotein fractions were then collected, desalted (final salt concentration < 5 mM), and concentrated using Amicon Ultra-0.5 mL centrifugal filter units (10 KDa MWCO, Millipore). Protein concentration was determined using DC Protein Assay Kit (Bio-Rad), according to the manufacturer's instructions, using bovine serum albumin as standard. Aliquots of 500 *μ*g (as protein) of LDL and 300 *μ*g (as protein) of VLDL were delipidated by adding ice-cold tri-n-butylphosphate : acetone : methanol (1 : 12 : 1) as reported by Mastro and Hall [24]. Delipidated fractions were resolubilized with repeated boiling and sonication passages in 10 mM Tris buffer containing 4% CHAPS (w/v) and 1% DTT (w/v). After cooling to room temperature, samples were diluted with a solution containing 8 M urea, 4% CHAPS, 1% DTT, and 0.4% carrier ampholytes and subjected to further sonication passages followed by 2-DE. 50 *μ*g (as protein) of native HDL fraction were solubilized for 2-DE analysis as reported above for LDL and VLDL delipidated fractions.

### 2.3. 2-DE Analysis

2-DE was performed as previously described [20]. Briefly, samples were applied to 70 mm IPG strips (pH 4–7, Bio-Rad), by overnight rehydration loading at 20°C, and subsequently focused at 50 *μ*A/IPG strip for 22 kVh at 20°C. After focusing, proteins were in-gel reduced by incubating IPG strips with a 50 mM Tris buffer containing 6 M urea, 30% glycerol (v/v), 3% SDS (w/v) and 1% DTT (w/v), followed by in-gel alkylation, using the same solution containing 2.5% iodoacetamide (w/v) in place of DTT. Each step was performed keeping strips under continuous shaking for 15 minutes. Then, IPG strips were sealed, with 0.5% low melting point agarose (w/v) in SDS running buffer, at the top of second dimension gels (8 cm × 7 cm × 0.1 cm). SDS-PAGE was carried out on 15% T/3% C polyacrylamide gels at 50 V for 15 minutes and subsequently at 150 V for about 90 minutes. Then, gels were fixed in 30% ethanol (v/v), 2% phosphoric acid (v/v) for 1 h, washed twice in 2% phosphoric acid (v/v) solution for 10 minutes, equilibrated in 18% ethanol (v/v) and 2% phosphoric acid (v/v), and 15% ammonium sulfate (w/v) solution for 30 minutes, and stained in the same solution added with 0.02% Coomassie Brilliant Blue G-250 (w/v) for 48 h. Gel images were acquired by using GS-800 calibrated densitometer (Bio-Rad) at 36.3 *μ*m resolution. Image analyses were performed using PD-Quest 2-D analysis software V 8.0.1 (Bio-Rad). Each spot was assigned a relative value corresponding to the single spot volume compared to the volume of all spots in the gel, following background subtraction and normalization between gels. In particular, in the adopted normalization method, the raw quantity of each spot in a member gel was divided by the total quantity of all the spots in that gel that have been included in the Master.

### 2.4. In-Gel Digestion and MALDI-TOF MS Analysis

Spots of interest were excised with sterile pipette tips, destained with a solution containing 2.5 mM NH_4_HCO_3_ and 50% acetonitrile (v/v), dehydrated with 100% acetonitrile, and dried at room temperature before proteolytic treatment. Tryptic digestion was performed by incubating dried spots in 5 mM NH_4_HCO_3_ buffer containing 10 ng/*μ*L trypsin overnight at 37°C. The resulting peptides were mixed with an equal volume of *α*-cyano-4-hydroxycinnamic acid saturated solution (40% acetonitrile (v/v) and 0.1% trifluoroacetic acid (v/v)) and applied as a microcrystalline thin film onto a stainless steel 96-spot MALDI target. Mass analyses were performed using a MALDI micro-MX-mass-spectrometer (Waters), according to the tuning procedures suggested by the manufacturer. Peak lists were generated using Protein Lynx Global Server v.2.2.5 (Waters) and searched against the Swiss-Prot human database (version 57.4) using Mascot (http://www.matrixscience.com). Research parameters included taxa (*Homo sapiens*), trypsin digest, monoisotopic peptide masses, iodoacetamide modifications, one missed cleavage by trypsin, and a mass deviation of 50 ppm. Only protein identifications with significant Mascot scores (*P* < 0.05) were taken into consideration.

### 2.5. Western Blotting Analysis

Western blotting analyses on proteins resolved by SDS-PAGE were performed as previously described [20]. Briefly, resolved proteins were electroblotted onto Hybond-P PVDF membranes (GE Healthcare) at 250 mA for 1.5 hours. Afterwards, membranes were incubated with blocking solution (PBS, 0.1% Tween-20, 3% nonfat dry milk) for 1 h at room temperature followed by overnight incubation at 4°C with a monoclonal anti-SAA antibody (Abcam, Ab81483) diluted 1 : 1000 or a polyclonal anti-apo AI antibody (Millipore, AB740) diluted 1 : 8000 with blocking solution. Then, after 30 minutes washing (PBS, 0.1% Tween-20), membranes were incubated with HRP-conjugated secondary antibody solution for 1 h at room temperature. Following further membrane washing, proteins were revealed by enhanced chemiluminescence using ChemiDoc XRS System (Bio-Rad). Densitometric analysis was performed using Quantity One 4.6.3 software (Bio-Rad).

### 2.6. Statistical Analysis

Student's *t*-test for unpaired samples was performed to compare exchangeable apolipoproteins expression in each purified lipoprotein fraction among atherosclerotic patients and healthy controls, using the software package Sigma Stat 3 (Systat Software). Significance was set at *P* < 0.05.

## 3. Results

### 3.1. Mapping of Exchangeable Apolipoproteins from VLDL, LDL, and HDL Fractions

2-DE combined with Mass Spectrometry was applied to identify VLDL, LDL, and HDL apolipoproteins from patients undergoing carotid endarterectomy and healthy normolipidemic subjects. According to echo-Doppler analysis, patients selected for surgery had either a high-grade carotid stenosis (>70%) or an ulcerated lesion. Since apo B100 is the most abundant apolipoprotein of both LDL and VLDL, representing up to 95% of their protein moiety, large amounts of LDL and VLDL were processed in order to allow the detection and identification also of less abundant apolipoproteins. Furthermore, due to the high lipid/protein ratio in both lipoprotein classes, a delipidation step was mandatory.

**Figure 1:**
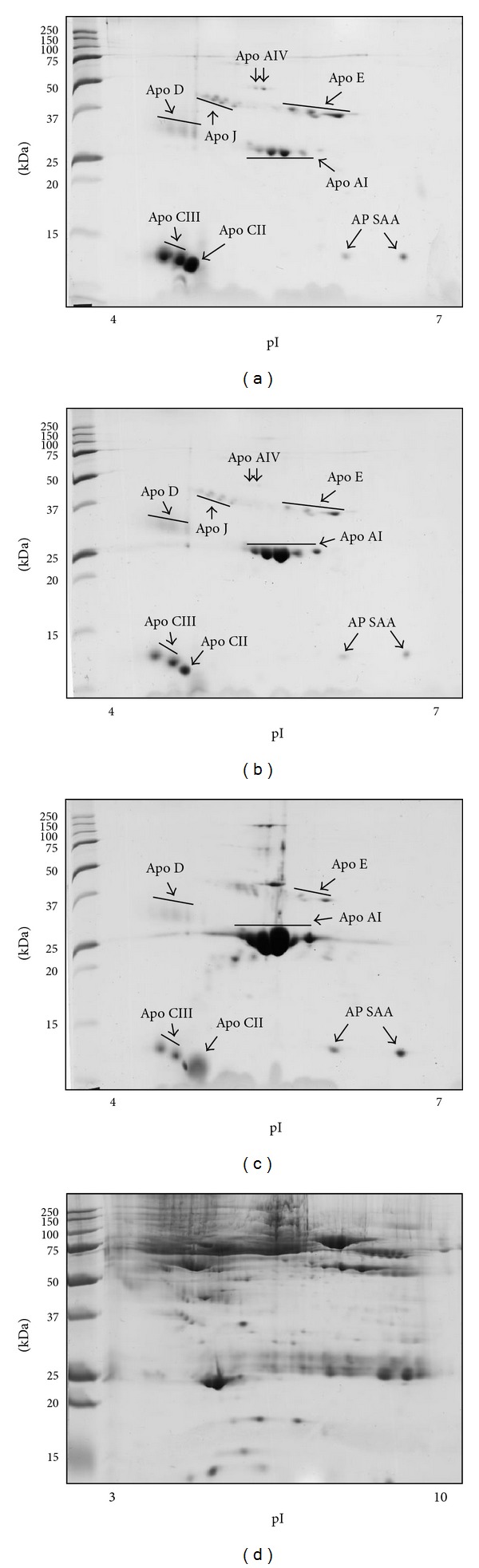
Protein expression patterns of VLDL (a), LDL (b), HDL (c) fractions, and plasma (d). Exchangeable apolipoproteins from purified lipoprotein fractions were resolved by isoelectric focusing on pH 4–7 IPG strips followed by SDS-PAGE on 15% T and 3% C gels (a–c). Plasma proteins were resolved by isoelectric focusing on pH 3–10 IPG strip followed by SDS-PAGE on 10% T and 3% C gel (d). Protein spots identified by MALDI-TOF MS analysis are marked.

Reproducible 2-DE maps of exchangeable apolipoproteins from VLDL, LDL, and HDL fractions were obtained (Figure 1). The adopted fractionation procedure, which included an isopycnic gradient ultracentrifugation followed by an additional washing step, allowed high purification yields of all lipoprotein fractions. As a matter of fact, no albumin or additional plasma proteins were detectable in 2-DE gels after Coomassie Brilliant Blue G-250 staining. Moreover, western blotting analysis of the HDL fraction did not reveal any apolipoprotein B-100 contamination (data not shown).

Almost all the resolved protein spots were identified by peptide mass fingerprinting (Table 2). In more detail, we identified 2 isoforms of apo J, 2 isoforms of apo AIV, 5 isoforms of apo E, 6 isoforms of apo AI, 3 isoforms of apo D, 2 isoforms of acute phase serum amyloid A protein, 1 isoform of apo CII and 2 isoforms of apo CIII, in both VLDL and LDL fractions. Due to its high molecular mass (about 500 kDa) and hydrophobicity, apo B100 was not detectable in any VLDL or LDL 2-DE maps. Regarding to HDL, 3 isoforms of apo E, 6 of apo AI, 3 of apo D, 2 of AP SAA, 1 of apo CII, and 2 of apo CIII were identified.

### 3.2. Differential Apolipoproteins Expression Analysis

**Figure 2:**
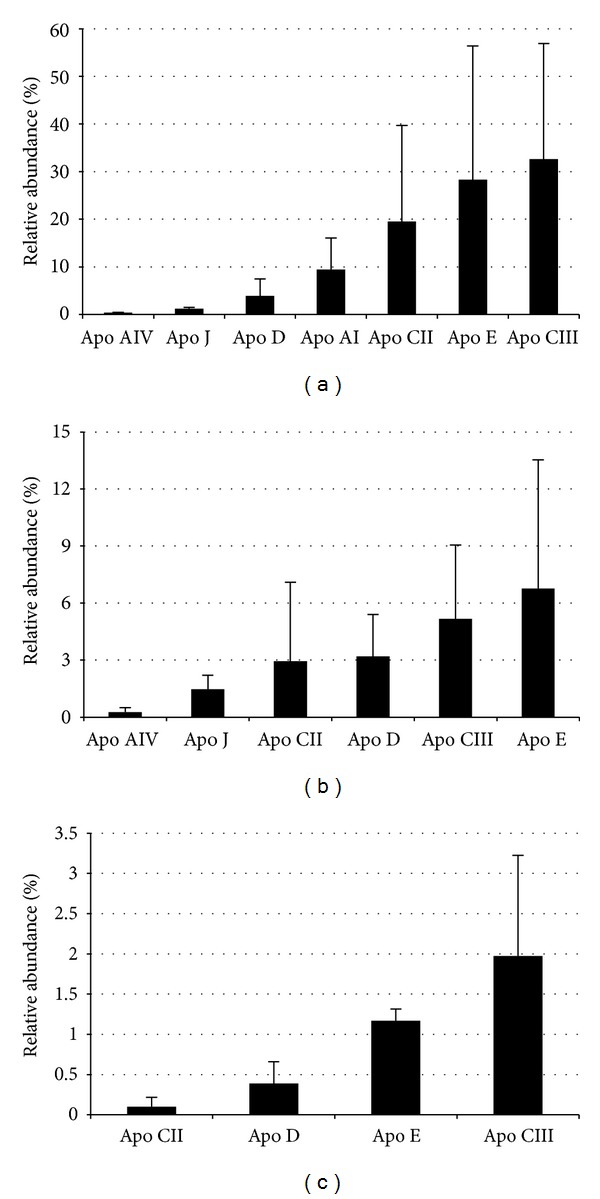
Relative amounts of exchangeable apolipoproteins in VLDL (a), LDL (b), and HDL (c) fractions representative of both patients and controls. Data were obtained by image analysis on 2-DE maps from all examined pooled plasma samples (4 obtained from patients and 4 from healthy controls) using PD-Quest analysis software V 8.0.1. Because of its high percentage in respect to the other apolipoproteins, apo AI was omitted in the panels corresponding to LDL (b) and HDL (c) fractions, being 72.9 ± 9.8% and 91.5 ± 3.7%, respectively. Relative amount of AP SAA is reported in Figure 3. Data are expressed as mean ± SD.

**Figure 3:**
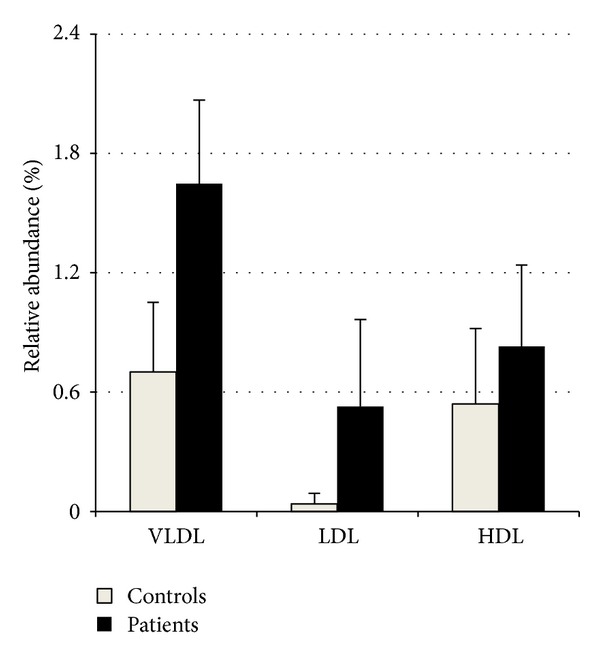
Relative amount of AP SAA in VLDL, LDL, and HDL fractions. AP SAA was found differentially expressed in all purified fractions in patients compared to controls (*P* = 0.001, *P* = 0.003, and *P* = 0.045, for VLDL, LDL, and HDL fractions, resp.). Data were obtained by image analysis on 2-DE maps from 4 pooled plasma samples obtained from patients and 4 from healthy controls using PD-Quest analysis software V 8.0.1. Data are expressed as mean ± SD.

**Figure 4:**
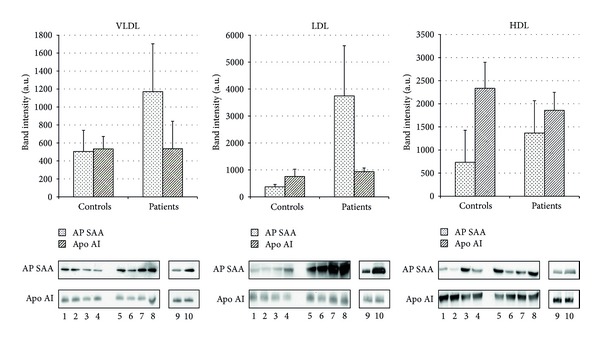
One-dimensional western blotting analysis. Bar charts reporting AP SAA and apo AI expression in VLDL, LDL, and HDL fractions isolated from 4 pooled plasma samples obtained from 57 controls (lanes 1–4) and 4 from 79 patients (lanes 5–8). Following data normalization for apo AI levels, AP SAA was confirmed as differentially expressed in all purified fractions from patients compared to controls (*P* = 0.03, *P* = 0.03, and *P* = 0.05, for VLDL, LDL, and HDL fractions, resp.). Results obtained on a randomly chosen sample from controls group (lane 9) and from patients group (lane 10) are shown. 10 *μ*g and 1 *μ*g of proteins were loaded for AP SAA and apo AI immunodetection, respectively.

Differential expression analysis was performed on VLDL, LDL, and HDL fractions from 4 pooled plasma samples of 79 patients having severe atherosclerosis and 4 pooled plasma samples of 57 healthy volunteers revealing no differences in the relative abundance of all identified apolipoproteins (Figure 2) with the exception of AP SAA that was more abundant (2.3-, 14.0-, and 1.5-fold in VLDL, LDL, and HDL fractions, resp.) in patients (*P* = 0.001, *P* = 0.003, and *P* = 0.045, for VLDL, LDL, and HDL fractions, resp.) (Figure 3). Differential AP SAA expression was confirmed by western blotting analysis on each lipoprotein fraction using apo AI levels for data normalization (*P* = 0.03, *P* = 0.03, and *P* = 0.05, for VLDL, LDL, and HDL fractions, resp.) (Figure 4). To further corroborate these preliminary observations, the same western blotting assay was performed on a randomly chosen sample from each group (Figure 4).

## 4. Discussion

By means of two-dimensional electrophoresis (2-DE) coupled with Mass Spectrometry (MS) analysis on plasma-purified VLDL, LDL, and HDL fractions we were able to identify acute phase serum amyloid A protein (AP SAA) as a potential marker of advanced carotid atherosclerosis.

The protein moiety of the different lipoprotein species is largely responsible for their functions, being involved in several metabolic events, such as recognition by specific receptors on cell surfaces or regulation of the activity of various enzymes [6]. Obtaining detailed information about apolipoprotein composition may contribute to revealing their role in atherogenesis. To date, only few studies have dealt with lipoproteomics in relation to atherosclerosis or atherosclerosis-related diseases, wih most of them being performed on HDL from coronary artery disease (CAD) patients [10–13].

This is the first report focusing on differential apolipoprotein expression in atherosclerotic patients undergoing carotid endarterectomy compared to healthy controls. As the adopted method is very labour intensive, we decided to set it up on randomly combined pooled plasma samples. In our opinion, this is the major limitation of the study, since each data reported represents an average value from 15 to 20 samples and a measure of how much variation or dispersion from the average exists is missing. On the other hand, this approach allowed for comparing levels of the exchangeable apolipoproteins in each lipoprotein fraction from a noteworthy number of patients and controls (79 patients and 57 controls). Indeed, the preliminary results obtained from differential protein expression analysis may represent a useful starting point for further, more specific analyses that could be carried out on each sample separately (e.g., ELISA on plasma samples and plaque extracts, and immunohistochemistry on endarterectomy specimens).

The apolipoprotein profile of VLDL, LDL, and HDL fractions, obtained from patients with advanced atherosclerosis was analysed, identifying 23 different protein isoforms by peptide mass fingerprinting. Moreover, we compared the obtained profiles with those from healthy volunteers.

The adopted purification procedure provided highly purified lipoprotein fractions as confirmed by the absence of plasma proteins as well as of apo B-100 in HDL (assessed by western blotting). Furthermore, due to the high number of resolved protein isoforms and the low limit of detection, 2-DE has proven to have high resolution and sensitivity.

Image analysis allowed for identifying AP SAA as differentially expressed in atherosclerotic patients. Its levels were higher in all lipoprotein fractions, increasing up to fourteenfold in atherosclerotic patients LDL fraction. Such evidence was confirmed by western blotting analysis on each lipoprotein purified fraction.

Serum amyloid A protein belongs to a family of acute-phase proteins, synthesized primarily in the liver, that circulate mainly associated to HDL. During the acute-phase reaction, plasma AP SAA concentration may increase up to 1000-fold due to an upregulation of its synthesis by inflammatory cytokines or peptide hormone signals [25]. Besides some evidence of an antiatherogenic role in promoting cholesterol efflux from cells [26–30], a plethora of studies have suggested proatherogenic effects for SAA, such as promotion of monocytes and neutrophils chemotaxis [31–33], macrophage foam cells formation [34], stimulation of proinflammatory cytokines secretion by monocytes-macrophages and lymphocytes [33, 35, 36], and induction of endothelial dysfunction [33, 37, 38]. Furthermore, SAA may potentiate prothrombotic events by inducing tissue factor expression [39]. A role for SAA in lipoprotein subendothelial retention has been postulated, since it is known that it could stimulate the synthesis of vascular proteoglycans with an increased LDL-binding affinity [40] and a proteoglycan binding domain on its carboxy-terminal region has been identified [41]. Furthermore, a study on *in vivo* murine model demonstrated that SAA deposited in atherosclerotic areas at all stages of lesion development and highly correlated with lesion area, apo AI area, apoB-100 area, and perlecan area [42]. Nevertheless, both protein localization [43] and mRNA expression [44] in human atherosclerotic plaques were demonstrated. SAA was also shown to increase in patients with atherosclerosis of the coronary and peripheral arteries [45–50]. Interestingly, Ogasawara et al. evidenced that LDL-associated SAA represents a marker of intravascular inflammation in patients with stable CAD, more sensitive than C-reactive protein, or free SAA [51]. Finally, a strong independent relationship between SAA and future cardiovascular events was demonstrated [52, 53]. All this evidence underlines not only the potential of this protein as a marker of advanced vascular disease but also the mechanisms by which SAA plays a direct role in atherosclerosis development.

Our results further support findings on proatherogenic effects of AP SAA and suggest a role of LDL as AP SAA carrier into the artery wall, where it could exert its noxious effects.

## 5. Conclusions

Our aim was to evaluate the apolipoprotein expression profiles in VLDL, LDL, and HDL fractions from patients undergoing carotid endarterectomy and healthy normolipidemic subjects by means of 2-DE coupled to Mass Spectrometry. By this approach, for the first time, AP SAA was identified as differentially expressed. Its levels were higher in all lipoprotein fractions, rising up to fourteen fold in LDL from atherosclerotic patients, indicating a potential association between AP SAA and the presence of advanced carotid lesions. Although preliminary, our findings are in accordance with a number of *in vitro* and *in vivo* evidence sources for a contributory role of this protein in atherogenesis. The adopted methodological approach was proven to be a powerful tool for identifying lipoprotein-associated markers in cardiovascular research. Further studies are in progress to evaluate the potential of LDL-associated AP SAA as a marker of carotid atherosclerosis as well as the effects of its increase in inducing inflammatory mechanisms underlying plaque development and progression/vulnerability.

## Figures and Tables

**Table 1:** Clinical characteristics and lipid levels of patients and controls.

Parameters	Patients (79)	Controls (57)
Age (years)*	69.2 ± 7.2	62.5 ± 23.9
Sex ratio, m/f	48/31	24/33
Symptomatic, %	40	—
Transient ischemic attack, %	28.6	—
Ictus, %	71.4	—
Diabetes, %	30.6	—
Hypertension, %	87.8	—
Dyslipidemic, %	83.7	—
Triglycerides, mg/dL*	123.9 ± 53.2	86.6 ± 28.7
Total cholesterol, mg/dL*	172.1 ± 43.4	175.7 ± 17.5
LDL cholesterol, mg/dL*	97.4 ± 37.8	92.6 ± 32.1
HDL cholesterol, mg/dL*	47.3 ± 13.4	50.1 ± 10.9

*Values are mean ± SD.

**Table 2:** MALDI TOF MS data obtained from triplicate experiments.

Apolipoprotein	No. of isoforms	Accession no.	Nominal mass	pI	Start	End	Observed^a^	Mr. (expt.)^b^	Mr. (calc.)^c^	Delta	Miss	Unique peptide^d^	Protein score^e^	Prot. Seq. Cov. (%)^f^
Apo AI	6	gi∣90108664	28061	5.27	1	10	1226.6349	1225.6276	1225.5364	0.0913	0	−.DEPPQSPWDR.V	209	48
					24	40	1815.9786	1814.9713	1814.8435	0.1278	1	K.DSGRDYVSQFEGSALGK.Q		
					28	40	1400.7913	1399.7840	1399.6620	0.1221	0	R.DYVSQFEGSALGK.Q		
					46	59	1612.9009	1611.8936	1611.7781	0.1156	0	K.LLDNWDSVTSTFSK.L		
					60	77	2202.2297	2201.2224	2201.1117	0.1108	1	K.LREQLGPVTQEFWDNLEK.E		
					62	77	1933.0461	1932.0388	1931.9265	0.1123	0	R.EQLGPVTQEFWDNLEK.E		
					62	83	2618.3394	2617.3321	2617.2660	0.0661	1	R.EQLGPVTQEFWDNLEKETEGLR.Q		
					97	106	1252.7203	1251.7130	1251.6136	0.0995	0	K.VQPYLDDFQK.K		
					97	107	1380.8340	1379.8267	1379.7085	0.1182	1	K.VQPYLDDFQKK.W		
					107	116	1411.7921	1410.7848	1410.6601	0.1247	1	K.KWQEEMELYR.Q		
					108	116	1283.7026	1282.6953	1282.5652	0.1301	0	K.WQEEMELYR.Q		
					119	131	1467.9192	1466.9119	1466.7841	0.1278	1	K.VEPLRAELQEGAR.Q		
					132	140	1152.6199	1151.6126	1151.6298	−0.0172	1	R.QKLHELQEK.L		
					161	171	1301.7749	1300.7676	1300.6411	0.1265	0	R.THLAPYSDELR.Q		

Apo AIV	2	gi∣178779	43358	5.22	32	45	1635.0740	1634.0667	1633.8311	0.2356	0	K.SELTQQLNALFQDK.L	180	44
					46	58	1407.9354	1406.9281	1406.7041	0.2240	0	K.LGEVNTYAGDLQK.K		
					60	70	1311.9370	1310.9297	1310.6983	0.2315	0	K.LVPFATELHER.L		
					93	103	1235.8699	1234.8626	1234.6670	0.1957	0	R.LLPHANEVSQK.I		
					115	123	1104.7449	1103.7376	1103.5611	0.1765	0	R.LEPYADQLR.T		
					150	167	1994.2684	1993.2611	1992.9864	0.2747	0	R.ENADSLQASLRPHADELK.A		
					170	180	1300.9045	1299.8972	1299.6783	0.2190	1	K.IDQNVEELKGR.L		
					192	200	1102.7498	1101.7425	1101.5666	0.1759	0	K.IDQTVEELR.R		
					192	201	1258.8846	1257.8773	1257.6677	0.2096	1	K.IDQTVEELRR.S		
					202	213	1350.8658	1349.8585	1349.6463	0.2122	0	R.SLAPYAQDTQEK.L		
					268	284	1928.2228	1927.2155	1926.9435	0.2720	0	K.SLAELGGHLDQQVEEFR.R		
					268	285	2084.2964	2083.2891	2083.0446	0.2445	1	K.SLAELGGHLDQQVEEFRR.R		
					286	296	1352.8851	1351.8778	1351.6520	0.2258	1	R.RVEPYGENFNK.A		
					297	308	1445.0040	1443.9967	1443.7868	0.2100	1	K.ALVQQMEQLRTK.L		
					309	325	1806.1775	1805.1702	1804.9108	0.2594	0	K.LGPHAGDVEGHLSFLEK.D		

Apo CII	1	gi∣14277770	8909	4.66	1	19	2203.1370	2202.1297	2202.0627	0.0670	0	−.TQQPQQDEMPSPTFLTQVK.E	82	68
					20	30	1286.6555	1285.6482	1285.5826	0.0656	0	K.ESLSSYWESAK.T		
					56	76	2233.2034	2232.1961	2232.1348	0.0613	0	K.STAAMSTYTGIFTDQVLSVLK.G		
					56	79	2548.3137	2547.3064	2547.2414	0.0650	1	K.STAAMSTYTGIFTDQVLSVLKGEE.−		

Apo CIII	2	gi∣186972736	8759	4.72	1	17	1907.0851	1906.0778	1905.8488	0.2290	0	−.SEAEDASLLSFMQGYMK.H	71	64
					25	40	1717.0728	1716.0655	1715.8438	0.2217	0	K.DALSSVQESQVAQQAR.G		
					41	51	1196.7615	1195.7542	1195.5874	0.1669	0	R.GWVTDGFSSLK.D		
					41	58	2076.2444	2075.2371	2075.0000	0.2371	1	R.GWVTDGFSSLKDYWSTVK.D		

Apo D	3	gi∣114034	21547	5.06	28	41	1657.8419	1656.8346	1656.7566	0.0780	0	K.CPNPPVQENFDVNK.Y	50	22
					46	60	1883.0264	1882.0191	1881.9261	0.0930	1	R.WYEIEKIPTTFENGR.C		
					52	60	1034.6072	1033.5999	1033.5193	0.0807	0	K.IPTTFENGR.C		
					152	164	1423.8282	1422.8209	1422.7354	0.0855	0	R.NPNLPPETVDSLK.N		

Apo E	5	gi∣178853	36185	5.81	34	43	1247.6713	1246.6640	1246.5691	0.0950	0	R.QQTEWQSGQR.W	90	30
					57	79	2730.4333	2729.4260	2729.3872	0.0388	0	R.WVQTLSEQVQEELLSSQVTQELR.A		
					94	108	1730.9484	1729.9411	1729.8370	0.1041	0	K.SELEEQLTPVAEETR.A		
					208	224	1752.9147	1751.9074	1751.9642	−0.0568	1	R.VRAATVGSLAGQPLQER.A		
					210	224	1497.9037	1496.8964	1496.7947	0.1017	0	R.AATVGSLAGQPLQER.A		
					259	269	1313.8163	1312.8090	1312.7099	0.0992	1	R.AKLEEQAQQIR.L		
					270	278	1033.5950	1032.5877	1032.5352	0.0525	0	R.LQAEAFQAR.L		
					281	292	1536.8112	1535.8039	1535.7079	0.0961	0	K.SWFEPLVEDMQR.Q		

Apo J	2	gi∣338305	36712	5.74	29	43	1873.0469	1872.0396	1871.8407	0.1989	0	R.QQTHMLDVMQDHFSR.A	80	35
					44	55	1393.8494	1392.8421	1392.6885	0.1536	0	R.ASSIIDELFQDR.F		
					60	75	2000.1421	1999.1348	1998.9588	0.1760	0	R.EPQDTYHYLPFSLPHR.R		
					168	183	1762.9714	1761.9641	1761.8203	0.1438	0	R.EILSVDCSTNNPSQAK.L + Carbamidomethyl (C)		
					187	197	1288.7798	1287.7725	1287.6306	0.1419	0	R.ELDESLQVAER.L		
					247	269	2314.3184	2313.3111	2313.1700	0.1411	0	R.VTTVASHTSDSDVPSGVTEVVVK.L		
					270	286	1874.1448	1873.1375	1872.9833	0.1542	0	K.LFDSDPITVTVPVEVSR.K		

AP SAA	2	gi∣225986	11675	5.89	2	15	1550.6927	1549.6854	1549.7202	−0.0347	0	R.SFFSFLGEAFDGAR.D	70	60
					26	39	1670.7418	1669.7345	1669.7848	−0.0503	1	R.EANYIGSDKYFHAR.G		
					48	62	1456.6875	1455.6802	1455.7106	−0.0304	0	R.GPGGVWAAEAISDAR.E		
					68	87	2177.8303	2176.8230	2176.9562	−0.1332	0	R.FFGHGAEDSLADQAANEWGR.S		

^a^ *m*/*z* value of the observed peak in MS analysis; ^b^molecular weight expected for peptide; ^c^molecular weight calculated; ^d^unique peptides identified in the spot; ^e^Mascot score; ^f^percentage of protein coverage.
